# *Cytokinin Oxidase/Dehydrogenase 1* (*FvCKX1*) Coordinates Receptacle Growth and Achene Maturation in Strawberry

**DOI:** 10.3390/plants15081171

**Published:** 2026-04-10

**Authors:** Yunhe Tian, Luyan Ren, Ziyin Zhu, Zhiyun Chen, Yue Yuan, Yahui Lv, Wei Xin, Chutian Wu, Jun Ma, Jun He, Juncheng Lin, Yanlin Liu, Tongda Xu, Wenxin Tang

**Affiliations:** 1 College of Life Sciences, and Haixia Institute of Science and Technology, Fujian Agriculture and Forestry University, Fuzhou 350002, China; yunhe_tian13@163.com (Y.T.); 18650097006@163.com (L.R.); zhuziyin2022@163.com (Z.Z.); caocaochen0120@163.com (Z.C.); yue-yuan@fafu.edu.cn (Y.Y.); lvyh1122@163.com (Y.L.); xinwei@wuyiu.edu.cn (W.X.); 3255302034@stu.fafu.edu.cn (C.W.); junma@fafu.edu.cn (J.M.); 000q820003@fafu.edu.cn (J.H.); linjc@fafu.edu.cn (J.L.); liuyanlin@fafu.edu.cn (Y.L.); 2 College of Horticulture, Fujian Agriculture and Forestry University, Fuzhou 350002, China; 3 College of Ecology and Resources Engineering, Wuyi University, Wuyishan 354300, China

**Keywords:** strawberry, achene, receptacle, ripening transition, cytokinin oxidase/dehydrogenase, abscisic acid

## Abstract

The coordinated development of achenes and receptacles in strawberry is critical for seed dispersal and fruit quality, yet the underlying molecular mechanisms remain poorly characterized. Utilizing RNA-seq analysis during the ripening transition stage, we identified pronounced transcriptomic divergence between achenes and receptacles, with receptacles exhibiting more dynamic gene expression shifts. Intriguingly, a substantial subset of differentially expressed genes (DEGs) displayed antagonistic expression patterns between these tissues, including the cytokinin degradation gene *cytokinin oxidase/dehydrogenase 1* (*FvCKX1*), which was highly expressed in both tissues but with opposing temporal trends. Functional interrogation via transient silencing and overexpression revealed a tissue-specific regulatory role for *FvCKX1*. RNA interference (RNAi) suppression of *FvCKX1* significantly enhanced receptacle expansion but delayed achene maturation, whereas overexpression inhibited receptacle growth while accelerating achene ripening. Abscisic acid (ABA), which positively regulates fruit enlargement, was elevated in *FvCKX1* RNAi receptacles and notably reduced in overexpression fruits, indicating that FvCKX1 might negatively modulate ABA synthesis during strawberry fruit development. Our results demonstrate that FvCKX1 may function as a key regulator mediating the coordinated development between achenes and receptacles in strawberry.

## 1. Introduction

Fruits have evolved to provide nourishment, protection, and facilitate seed dispersal [1,2,3,4]. The regulation of fruit growth and ripening is largely dependent on the growth and development of seeds. In nature, fruit growth is initiated in response to fertilization and ripening commences after embryo maturation. For fleshy fruits, ripening is characterized by a sweet taste, vibrant color, and succulent and flavorful tissue to attract vertebrates for consumption. This ultimately leads to seed dispersal as they are carried away for dispersal before being deposited as nutrients to germinate elsewhere [5,6,7]. Therefore, the coordinated development of the achene and receptacle is crucial for seed dispersal and progeny reproduction, yet the mechanism underlying this coordinated development remains unclear.

Fruit development can usually be divided into three distinct stages: fruit set, fruit growth, and fruit maturation [8,9,10]. Phytohormones and their downstream transcription factors play a vital role in all the stages of fruit development [1,6,11,12,13]. Auxin and gibberellins, in particular, play crucial roles in fruit initiation and enlargement, with the interaction of AUXIN RESPONSE FACTORS (ARFs) and gibberellin acid (GA) signaling factor aspartic acid–glutamic acid–leucine–leucine–alanine (DELLA) serving as the primary mediator [14,15,16,17]. The FRUITFULL (FUL) MADS-box transcription factor, which interacts with ARF6 and ARF8, promotes fruit elongation [18,19,20,21]. Meanwhile, the MADS-box transcription factor SEEDSTICK (STK) regulates fruit elongation by directly regulating cytokinin degradation and indirectly regulating the expression of FUL [21,22,23,24].

Cytokinins (CKs) are phytohormones that regulate cell division and play crucial roles during fruit set and early fruit development [22,25,26,27]. Natural cytokinins can be classified into two major groups: isoprenoid CKs (more abundant and biologically active) and aromatic CKs (less common). Among the isoprenoid CKs, isopentenyladenine (iP) and trans-zeatin (tZ) are the primary bioactive forms, while cis-zeatin (cZ) and dihydrozeatin (DZ) are less active [28]. CK biosynthesis is controlled by isopentenyl transferase (IPT), the key rate-limiting enzyme, whereas cytokinin oxidase/dehydrogenase (CKX) irreversibly degrades CKs and is the only known enzyme specifically involved in their catabolism [28,29]. During fruit ripening stages, cytokinin transforms into a “speed reducer”, which inhibits the CKX activity then delays chlorophyll degradation [30], or combines with ethylene then delaying tomato fruit color transition and softening [31].

Ethylene and Abscisic acid (ABA) are the two key phytohormones that regulate late-stage ripening of climacteric and non-climacteric fruits, respectively [1,6,32,33]. Mutants, such as *RIPENING INHIBITOR* (*rin*) and *COLORLESS NON-RIPENING* (*cnr*), have facilitated the identification of key regulatory factors, such as *MADS-RIN* and *CNR*, which influence ethylene production in tomato [34,35]. Recent studies have highlighted the accumulation of ABA in non-climacteric fruits during the ripening phase, as well as the role of ABA in repressing the expression of *CYP707A4a*, which catalyzes ABA catabolism [36]. Additionally, the expression of *CYP707A4a* is induced by auxin and GA during early developmental stages, although further research is required to elucidate the regulatory mechanism of ripening of non-climacteric fruits.

Some evidence suggests that the development of seeds and fruits are not independent of each other but involve signal exchange and coordinated development. Auxin and GA, which are synthesized in the fertilized seeds, act as growth signals that stimulate fruit growth [37,38]. Moreover, the number of seeds within a fruit influences its ripening. However, the mechanism underlying the interplay between seeds and fruit development remains largely unknown. Strawberry fruit, with its unique attachment of achenes to the surface of an enlarged receptacle, provides a valuable research model to explore this phenomenon, as the role of seeds in fruit development and ripening can be easily observed [39,40,41,42]. Previous studies have shed some light on this relationship, but the precise mechanisms involved require further study.

The coordinated development of achenes and receptacles is critical for strawberry fruit enlargement and seed dispersal. Although several studies have suggested that hormones such as auxin and GA are involved in the coordinated development of achenes and receptacles at early stages in strawberry [36,43,44], the coordinated regulatory mechanism between achenes and receptacles remains largely unknown during other developmental stages, especially the ripening transition phase that determines fruit size and seed maturation. To identify the coordinated regulatory factors involved in the crosstalk between achenes and receptacles during the ripening transition phase, we evaluated the transcriptional regulation of the achene and receptacle during the ripening transition phase in strawberry fruits. *Cytokinin oxidase/dehydrogenase 1* (*FvCKX1*) was identified as a gene that showed differential regulation during the ripening transition phase, with varying expression patterns observed between the achene and receptacle. We found FvCKX1 played a key role in the transition from the growth phase of achene and receptacle, and also influenced ABA accumulation in the receptacle of *Fragaria vesca*. This gene may coordinately regulate achene and receptacle development by dynamically modulating cytokinin (CK) and abscisic acid (ABA) accumulation. These findings provide a better understanding of the mechanisms underlying fruit development and ripening, while also laying the foundation for future functional verifications.

## 2. Results

### 2.1. Differential Growth Phases of Receptacle and Achene in Strawberry Fruit

**Figure 1:**
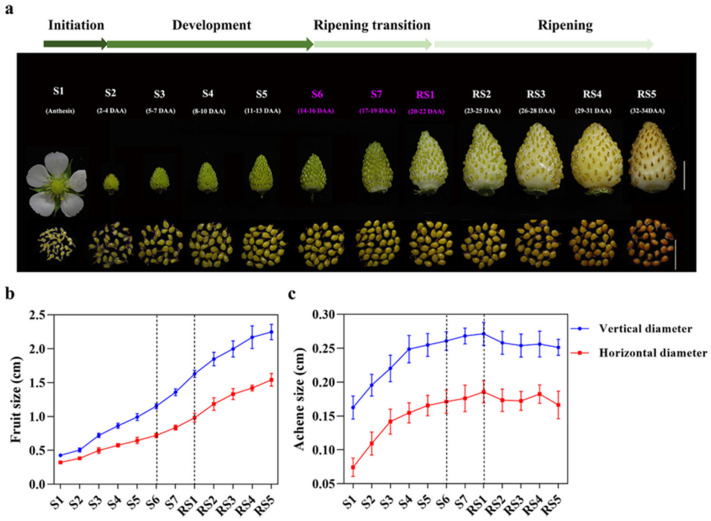
Differential growth phases between receptacles and achenes during strawberry fruit development. (**a**) Morphological characterization of strawberry fruits at 12 developmental stages. The achenes were separated from fruits in the corresponding stages. S1 to S2 are initiation stages, S2 to S6 are development stages, the fruit ripening transition occurs from S6 to RS1, RS1-RS5 are fruit ripening stages. S: developmental stage; RS: ripening stage. Scale bar = 1 cm. (**b**,**c**) Quantification of fruit and achene dimensions (vertical and horizontal diameter). Error bars indicate standard deviation (SD). *n* = 15–20 for (**b**). *n* = 60 for (**c**).

The development of strawberry fruit (achenes and receptacles) was divided into three phases and 12 stages [36], comprising early fruit development stage (S, S1–S6), ripening transition stage (S7-RS1) and ripening stage (RS, RS2-RS5). Based on the growth curves of receptacles and achenes, the achene and receptacle showed different developmental patterns during the entire fruit development (Figure 1). Receptacles continued to expand from S1 to RS5, and the expansion rate peaked at the stage of ripening transition (S7-RS1). After RS2, the expansion rate slowed down and the fruit firmness decreased (Figure 1a,b). Achenes exhibited peak expansion rate from S1 to S4 (Figure 1c) and then entered the pre-maturation (S5) and maturation (S6) stages [36]. Therefore, achenes rapidly expanded and matured earlier than receptacles during the entire strawberry development. The S6-RS1 period might be a critical period determining the final size and maturity of strawberry fruit.

### 2.2. Global Gene Expression in the Achene and Receptacle During Fruit Ripening Transition

**Figure 2:**
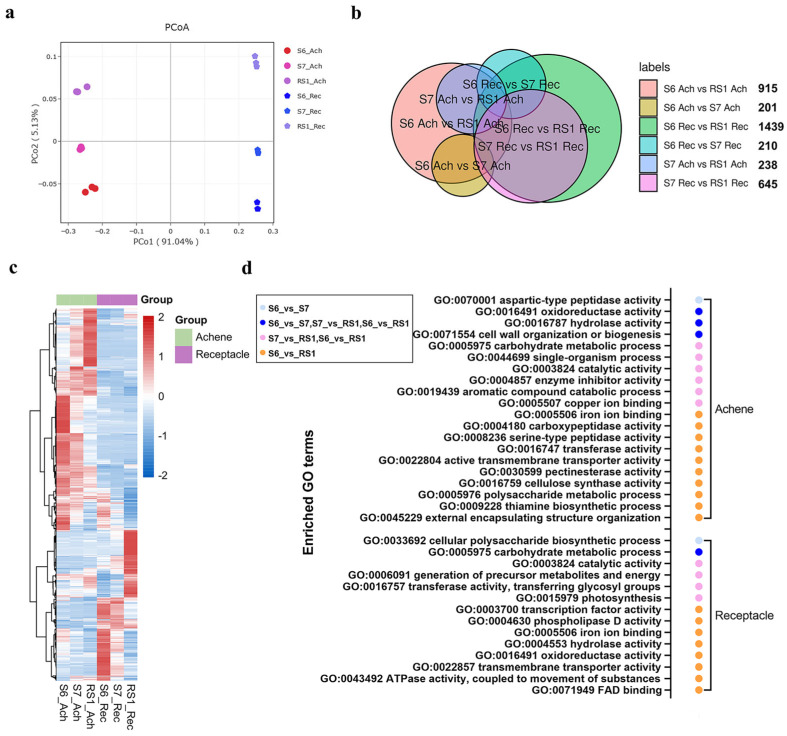
Transcriptional regulation during fruit ripening transition. (**a**) Principal coordinate analysis (PCoA) of transcriptomes across developmental stages. The whole transcription data were used, and X-ray and Y-ray show the distance between each sample. Ace, achene; Rec, receptacle. (**b**) Venn diagram depicting unique and shared differentially expressed genes (DEGs) between achene and receptacle tissues during ripening transition. Numerals indicate DEG counts. (**c**) An overview of the gene expression profiles of the 2412 DEGs identified in achenes and receptacles during S6-RS1 transition. *p* < 0.05, |log2(Fold change)| > 1.5. Expression values were calculated as Log2(value + 10^−6^). (**d**) The enriched Gene Ontology (GO) terms identified in the different comparing groups for achenes and receptacles, respectively. Legend on the left showed the coordinate comparing groups. *p* < 0.05.

To identify the key genes that regulate fruit ripening transition, receptacles and achenes from the S6, S7, and RS1 stages were separately sampled for RNA-seq analysis. Clean bases for each sample were obtained with an efficiency rate of over 92%, and counts of gene expression levels were generated using featureCounts. Subsequently, PCoA was conducted to explore the global relationship among samples. The results revealed a significant difference in overall gene expression between achenes and receptacles. However, both organs exhibited a similar trend of progressively increasing differences from S6 to RS1, suggesting that numerous genes might be activated during the ripening transition, especially in the receptacle (Figure 2a). The global gene expression also exhibited notable changes across different organs (receptacles and achenes) and stages (S6, S7, and RS1), with greater expression changes observed from S7 to RS1 than from S6 to S7 (Figure 2a).

Venn analysis was employed to compare unique and shared differentially expressed genes (DEGs) between achene and receptacle tissues during the ripening transition. Consistent with the PCoA results, DEG profiling revealed pronounced transcriptomic divergence between the two tissues, with minimal overlap in their respective DEG sets (Figure 2b and Figure S1). There were more DEGs from S7 to RS1 than from S6 to S7, especially in receptacles. In receptacles, 210 DEGs were identified between stages S6 and S7, while a greater number of DEGs (645) were discovered between stages S7 and RS1. In achenes, 201 DEGs were detected between stages S6 and S7, while 238 DEGs were observed between stages S7 and RS1. Furthermore, the overall number of DEGs across different stages of the receptacle tissue was higher than that of achenes, particularly during the maturation transition from S7 to RS1, indicating that receptacles may undergo more biological events during the S6 to RS1 stages (Figure 2b). Heat map analysis of the DEGs in both achenes and receptacles revealed marked differences in gene expression between the two tissues, with more pronounced changes in expression levels of DEGs from S7 to RS1 in receptacles than in achenes (Figure 2c).

Further Gene Ontology (GO) functional analysis revealed that the differentially expressed genes in achenes were involved in 20 biological processes (20 GO terms; Figure 2d). Among these GO terms, aspartic-type peptidase activity was specifically enriched in S6-vs-S7. 3 GO terms (oxidoreductase activity, hydrolase activity, cell wall organization or biosynthesis) were shared among all three groups. Six GO terms were significantly regulated in S7-vs-RS1 and S6-vs-RS1, and 10 GO terms were specifically enriched in S6-vs-RS1. The metabolic process of aromatic compound catabolic process was induced at RS1. In receptacles, although more DEGs were found, relatively few GO terms (14) were enriched. GO:0033692 cellular polysaccharide biosynthetic process was identified only in S6-vs-S7. The three groups shared the GO:0005975 carbohydrate metabolic process. GO:0015979 photosynthesis, GO:0006091 generation of precursor metabolites and energy, GO:0016757 transferase activity, transferring glycosyl groups, and GO:0003824 catalytic activity seemed differentially regulated at RS1 comparing with S6 or S7. Additional molecular functions were differentially regulated in S6-vs-RS1 including GO:0003700 transcription factor activity, GO:0004630 phospholipase D activity, GO:0022857 transmembrane transporter activity, etc., (Figure 2d).

Among the DEGs, a large number of genes related to fruit development were identified. Fruit development involves multiple biological processes, five of which are relatively well studied, including cell wall polysaccharides and enzymes, cell proliferation and size, photosynthesis, secondary metabolism and fruit development [2,7]. We conducted a detailed analysis of the expression changes of these five biological process-related genes in strawberry receptacles and achenes from S6 to RS1 (Figure S2). The results showed that most of the genes related to these five biological processes had a clear trend of increasing or decreasing expression in the receptacle. Most of the genes associated with cell wall polysaccharides and enzymes were upregulated from S6 to RS1. Most of the genes related to cell proliferation and size, photosynthesis and secondary metabolic were downregulated from S6 to RS1 (Figure S2). In achenes, only half of these genes were differentially expressed, and the trend was not obvious (Figure S2).

The regulatory mechanisms of fruit development have been elucidated in *Arabidopsis* through the identification of MADS-box transcriptional factors *SEEDSTICK* (*STK*) and *FRUITFULL* (*FUL*), which control fruit size via both auxin and cytokinin (CK) pathways. The CK levels accumulated during fruit elongation were found to be important for this process [45]. Our transcriptional data revealed differential regulation of four genes involved in this pathway in both the achene and receptacle. *STK* and *cytokinin oxidase/dehydrogenase 5* (*CKX5*) exhibited relatively high expression levels in the achene, while FUL was highly expressed in the receptacle. Notably, *FvCKX1*, which encodes cytokinin dehydrogenase, showed relatively high expression levels in both the achene and receptacle. Interestingly, during the transition from stage S6 to RS1, *FvCKX1* exhibited divergent expression patterns in these two interconnected fruit tissues (Figure S2).

### 2.3. FvCKX1 Has Opposite Dynamics in Achene and Receptacle During Ripening Transition

**Figure 3:**
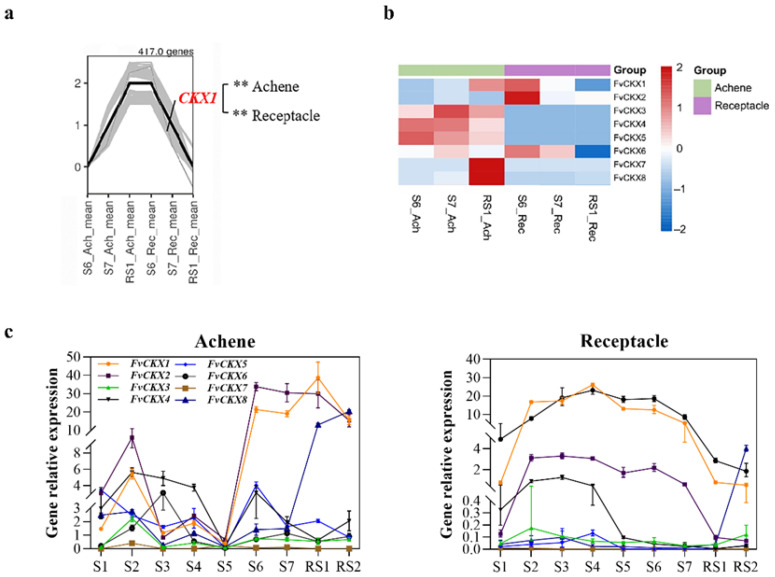
Cytokinin regulates the growth of achenes and receptacles during fruit ripening transition in *Fragaria vesca*. (**a**) *FvCKX1* was significantly regulated in achenes and receptacles, as identified in the gene co-expression module. In the module, gray lines represent the expression profiles of all individual genes and the black line represents the median expression profile. Statistical significance was analyzed using one-way ANOVA. **, *p* < 0.01. (**b**) *FvCKXs* expression pattern in the receptacle and achene during the S6-RS1 stages from RNA-seq data. (**c**) Expression profiles of *FvCKXs* genes across S1-RS2 stages in the achene and receptacle. Error bars represent SD of three independent replicates. Each gene was labeled with a corresponding color.

Upon analyzing the expression trends of DEGs from S6 to RS1 in achenes and receptacles, we discovered that 284 genes were upregulated in achene and downregulated in receptacle (Figure 3a). These genes might play opposite roles in regulating the development of receptacles and seeds, thereby coordinating their development. Among these genes, *FvCKX1*, a gene related to the degradation of cytokinin, has been reported through its orthologs to play an important role in regulating fruit development [46,47,48]. Expression pattern analysis of *FvCKX1* and other members of this gene family revealed that *FvCKX3,4,5* and *FvCKX7,8* had relatively high expression in achenes but low expression in receptacles. *FvCKX2* was highly expressed in receptacles but low in achenes. *FvCKX6* had a high transcription level in both achenes and receptacles but had no change in expression level from S6 to RS1 in achenes. Compared to *FvCKX2-8*, *FvCKX1* not only showed divergent trends in S6-RS1 but also had relatively higher expression levels in both achenes and receptacles (Figure 3b,c). Therefore, we selected *FvCKX1* as a candidate gene for simultaneous regulation of receptacle and achene development and further functional verification.

### 2.4. Transient Knockdown of FvCKX1 Promotes Receptacle Growth and Delays Achene Maturation

**Figure 4:**
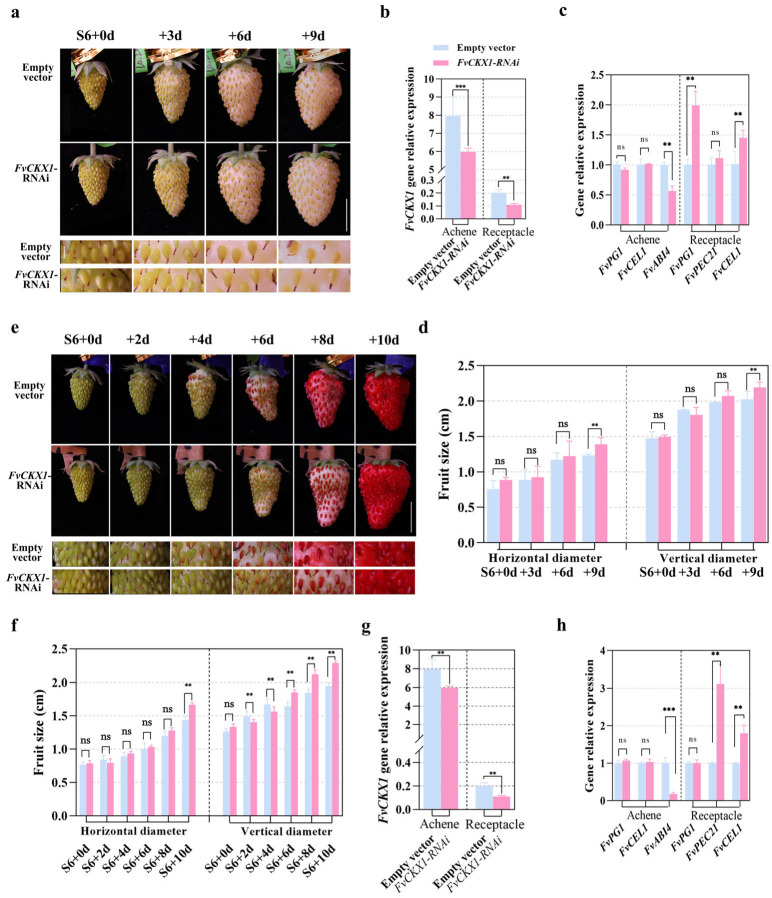
*FvCKX1* RNAi promotes the growth of receptacles and suppresses the maturity of achenes. (**a**) Transient knockdown of *FvCKX1* in S6-RS2 promotes receptacle growth while inhibits achene senility. The *FvCKX1*-RNAi construct and empty vector control were separately introduced into strawberry fruits at stage S6 via *Agrobacterium*-mediated transient transformation The transgenic fruits were analyzed every three days. Scale bar = 1 cm. Five fruits from three independent plants were observed. Three biological repeats were performed. (**b**) The transcript levels of *FvCKX1* in *FvCKX1*-RNAi receptacles and achenes during the S6 to RS2 stages. Error bars represent SD of three independent replicates. (**c**) Expression patterns of *FvPG1*, *FvPEC21* and *FvCEL1* in *FvCKX1*-RNAi fruits. Error bars represent SD of three independent replicates. (**d**) Quantification of fruit size variation in *FvCKX1*-RNAi fruits along with fruit development. Error bars represent SD of five fruits. (**e**) Expansion of the receptacle was promoted by *FvCKX1*-RNAi in red-type *Fragaria vesca*. The *FvCKX1*-RNAi construct and empty vector control were separately introduced into strawberry fruits at stage S6 via *Agrobacterium*-mediated transient transformation. Fruit growth was characterized every two days. Scale bar = 1 cm. (**f**) The effect of transient downregulation of *FvCKX1* on fruit size (horizontal diameter and vertical diameter). Error bars represent SD of five independent replicates. Transcript level variations due to transient silencing of *FvCKX1* are shown in (**g**). Error bars represent SD of three independent replicates. (**h**) Regulation of expression levels of *FvPG1*, *FvPEC21* and *FvCEL1* in the red-type fruit of *FvCKX1*-RNAi. Error bars represent SD of three independent replicates. Significant differences in (**b**–**d**) and (**f**–**h**) were analyzed using one-way ANOVA. ***, *p* < 0.001; **, *p* < 0.01; ns, no significant.

In order to investigate the role of *FvCKX1* in fruit ripening, a transient RNAi approach was employed to knock down its expression in strawberry fruits. A 276 bp fragment of *FvCKX1* was introduced into the pK7WIWG2D RNAi destination vector, which contains a p35S promoter-driven eGFP and had been shown to work well in strawberry [49,50]. Agrobacterium-mediated transformation was used to deliver the FvCKX1-pK7WIWG2D construct into Yellow Wonder S6 fruits, and successful knockdown of *FvCKX1* was confirmed by qRT-PCR analysis (Figure 4b). Subsequently, fruit sizes at developmental stages S7, RS1, and RS2 were measured. The results showed that the knockdown of *FvCKX1* promoted the enlargement of receptacles in both vertical and horizontal dimensions (Figure 4a,d), and upregulated two out of three ripening marker genes (*FvPG1* and *FvCEL1*) in the transgenic receptacles (Figure 4c). In addition, the knockdown affected the color change in achenes, with the mature *FvCKX1*-knockdown achenes exhibiting a delayed color change and downregulation of *FvABI4* expression compared to the control. In order to better observe the color change in achenes, this result was further confirmed in the red strawberry cultivar Rugen, where the knockdown of *FvCKX1* led to the enlargement of receptacles and a clear delay in color change in achenes, accompanied by down-expression of *FvABI4* (Figure 4e–h).

### 2.5. Transient Overexpression of FvCKX1 Represses Receptacle Growth and Promotes Achene Maturation

**Figure 5:**
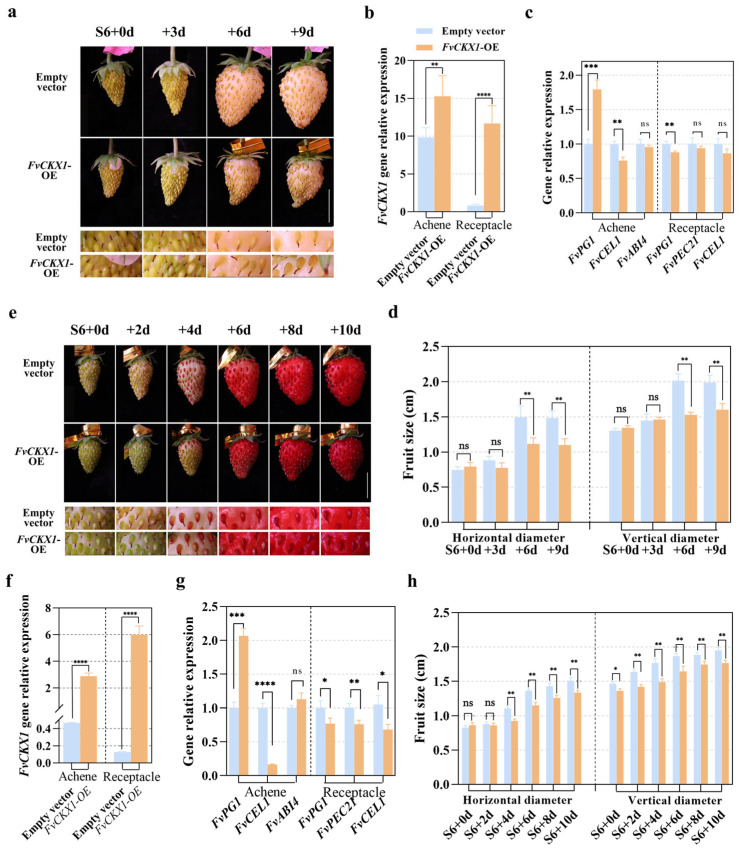
Transient overexpression of *FvCKX1* represses receptacle growth and promotes achene maturity. (**a**) Receptacle growth was defective for *FvCKX1*-OE S6-RS2 while achene senility was accelerated. The constructed *FvCKX1*-OE and empty vector were injected into the fruits at S6 through Agrobacterium. Phenotypes were characterized every three days. Scale bar = 1 cm. Five fruits from three independent plants were observed. Three biological repeats were performed. (**b**,**c**) qRT-PCR analysis showed a dramatic increase in *FvCKX1* expression (**b**) and *FvPG1*, *FvPEC21* and *FvCEL1* gene expression patterns (**c**) in the receptacle and achene for *FvCKX1*-OE fruits. Error bars represent SD of three independent replicates. (**d**) Statistical analysis of the fruit size variation in *FvCKX1*-OE fruits along with fruit development. Error bars represent SD, *n* = 5. Three biological repeats were performed. (**e**) Transient overexpression of *FvCKX1* reduces receptacle expansion for the red-type strawberry fruits. Scale bar = 1 cm. Gene expression levels for *FvCKX1*-OE were shown in (**f**). (**g**) The expression levels of *FvPG1*, *FvPEC21* and *FvCEL1* in the achene and receptacle. Error bars in (**f**,**g**) represent SD of three independent replicates. (**h**) The fruit size (horizontal diameter and vertical diameter) was analyzed for the red-type of *FvCKX1*-OE fruit. *n* = 5. Significant differences in (**b**–**d**,**f**–**h**) were analyzed using one-way ANOVA. ****, *p* < 0.0001; ***, *p* < 0.001; **, *p* < 0.01; *, *p* < 0.05; ns, no significant.

To further confirm the involvement of *FvCKX1* in fruit development, transient overexpression of *FvCKX1* was conducted in both white and red variants of strawberry (Figure 5b,f). The evaluation of fruit size and appearance demonstrated that *FvCKX1* overexpression significantly inhibited receptacle enlargement (Figure 5a,d,e,h). The maturation of achenes was then assessed through color alteration and the expression of maturation marker genes. In the white strawberry variant, Yellow Wonder, *FvCKX1* over-expression resulted in slightly darker-colored achenes in RS1 and RS2 in comparison to the empty vector control (Figure 5a), and *FvPG1* expression was upregulated in *FvCKX1* overexpressed achenes (Figure 5c). Similarly, in the red strawberry variant Rugen, *FvCKX1* overexpression led to a slight darkening of achenes 6–10 days after injection (Figure 5e), and *FvPG1* expression was upregulated in *FvCKX1* overexpressed achenes (Figure 5g). These findings indicate that *FvCKX1* may have an inhibitory effect on receptacle enlargement but a positive influence on achene maturation.

### 2.6. FvCKX1 May Negatively Regulate Abscisic Acid (ABA) Accumulation in Receptacles During Fruit Ripening Transition

CKXs are a class of cytokinin-degrading enzymes that may regulate fruit development by altering the balance of cytokinin content in strawberry achenes and receptacles. To test this hypothesis, we first measured the cytokinin content in achenes and receptacles at different stages of development and found that from the S6 to RS1 stages, the trans-zeatin content in achenes and receptacles exhibited opposite trends, with accumulation in achenes and decreasing levels in receptacles, a pattern opposite to the transcriptional levels of *FvCKX1* (Figure 3b and Figure S6). Over the course of fruit development, expression of the *FvCKX1* gene exhibited a negative correlation with trans-zeatin content in the strawberry fruit (Figure 3c and Figure S6a), indicating that *FvCKX1* may negatively regulate trans-zeatin levels in the fruit. Meanwhile, the effect of cytokinin on strawberry fruit development was identified through exogenous hormone treatments. The results showed that 100 μM TDZ (Thidiazuron, a well-known inhibitor of CKX [51]) significantly promoted the longitudinal but not lateral expansion of receptacles (Figure S4a,b), while 100 μM Lovastatin (cytokinin inhibitor) significantly inhibited the longitudinal but not lateral expansion of the receptacle (Figure S4c,d). Meanwhile, neither TDZ nor Lovastatin significantly affected achene size (Figure S4a,c). However, 100 μM TDZ inhibited the green-to-yellow transition of achene, while 100 μM Lovastatin promoted the green-to-yellow transition of achene (Figure S4a,c), suggesting that cytokinin might delay achene maturation. Red-fruit Rugen showed similar response to TDZ and Lovastatin to that of Yellow Wonder. The changes in achene from green to red were significantly delayed by 100 μM TDZ, while the green-to-red color transitions in achenes were accelerated by 100 μM lovastatin (Figure S5).

Based on the results above, it is reasonable to assume that *FvCKX1* may negatively regulate fruit enlargement by degrading cytokinin during strawberry ripening transition. In order to test this hypothesis, the content of trans-zeatin (a naturally occurring cytokinin) in transgenic strawberry fruits was determined using liquid chromatography–mass spectrometry (LC-MS). The results showed that trans-zeatin levels did not significantly change in the *FvCKX1*-OE receptacle, and a slight decrease in trans-zeatin content in the *FvCKX1*-RNAi receptacle compared to the control (Figure S6b).

**Figure 6:**
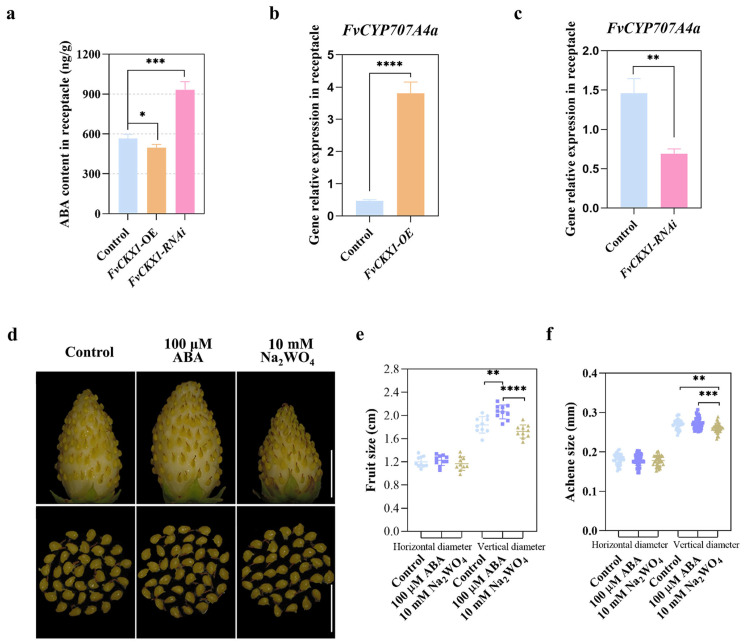
*FvCKX1* may negatively regulate ABA synthesis. (**a**) *FvCKX1* affected the variation in ABA content. Error bars represent SD of three independent repeats. Statistical significance was analyzed using one-way ANOVA. ***, *p* < 0.001; *, *p* < 0.05. (**b**,**c**) The expression levels of *FvCYP707A4a* in the receptacles of *FvCKX1* overexpression and *FvCKX1*-RNAi fruits. Significant differences were analyzed using Student’s *t*-test. ****, *p* < 0.0001; **, *p* < 0.01. (**d**–**f**) Phenotype (**d**) and size (**e**,**f**) were observed for fruit (including fruit and achene) growing under 500 µM ABA and 500 µM Na_2_WO_4_. Na_2_WO_4_, an ABA biosynthesis inhibitor. Scale bar = 1 cm. Error bars represent SD, *n* = 7–10 fruits or 60 achenes. Significant differences were analyzed using one-way ANOVA. ****, *p* < 0.0001; **, *p* < 0.01.

Notably, our investigation into hormone levels disclosed a significant decrease in abscisic acid (ABA) in *FvCKX1*-overexpressing receptacles, whereas ABA was significantly increased in *FvCKX1*-RNAi receptacles (Figure 6a). Correspondingly, the expression of *FvCYP707A4a*, a gene for ABA catabolism, was upregulated in the *FvCKX1* overexpressing receptacles and downregulated in the *FvCKX1*-RNAi receptacles of strawberry (Figure 6b,c), suggesting that *FvCKX1* may negatively regulate ABA accumulation in the receptacles. ABA is acknowledged to facilitate fruit ripening [36]. This inference was further supported by our experiments involving exogenous ABA and the ABA inhibitor Na_2_WO_4_. Specifically, 100 µM ABA treatment significantly promoted the horizontal and vertical enlargement of the strawberry fruit, while 10 mM Na_2_WO_4_ treatment significantly inhibited the fruit enlargement (Figure 6d–f). These outcomes imply that *FvCKX1* may hamper the enlargement of the receptacle by negatively modulating ABA biosynthesis.

## 3. Discussion

Strawberry seed dispersal mainly relies on consumption by birds and other animals. Before being consumed, strawberry seeds must first reach maturity, followed by the rapid enlargement and ripening of the receptacle, which attracts animals through its aroma and palatability. Therefore, the differential development of strawberry achenes and receptacles is critical for the reproductive success of strawberry progeny [5,52]. In *Fragaria vesca*, a notable distinction exists between the growth processes of achenes and receptacles, despite their physiological attachment [42,44,53]. Achenes exhibit earlier development compared to receptacles (Figure 1). The achenes reach full morphological development at stage S6 prior to entering maturation phase, whereas the receptacle initiates accelerated growth and cellular expansion at S6, culminating in ripening marked by anthocyanin accumulation from RS1 onward [36]. Differential gene expression patterns identified in achene and receptacle samples further support this distinction, with moderate regulation observed in achenes from S6 to RS1, while significant changes in gene expression occur in RS1 receptacles (Figure 2a,b). Notably, numerous highly expressed cell wall modification genes in receptacles exhibit significant differential regulation at RS1 (Figure S1). Additionally, pathways related to photosynthesis, precursor metabolite generation and energy production, and aromatic compound catabolism also exhibit distinct regulation during fruit ripening transition in achenes and receptacles (Figure 2). Thus, achene development precedes receptacle growth. During the early stage of strawberry fruit development, fruit growth is dominated by achene development and maturation, whereas receptacle growth is relatively suppressed. This inhibitory effect on receptacle growth is relieved once achene development is completed at the transition to fruit ripening.

Transcriptome analysis revealed a substantial subset of genes exhibiting opposite expression patterns in achenes versus receptacles during the ripening transition. Given the distinct developmental programs of these two tissues at this stage, we hypothesize that these genes may mediate their coordinated development. Notably, *CKX* (*cytokinin oxidase/dehydrogenase*) genes have been well-documented to regulate seed development [26,54,55]. Loss-of-function mutations in *Arabidopsis AtCKX3/5* or *OsCKX2* in rice or *GmCKX3/7/14* in soybean consistently increase seed yield [26,54,56]. In strawberry, *FvCKX1* displays not only antagonistic expression trends between the two tissues but also comparatively high abundance during the ripening transition. Preliminary evidence from transient modulation experiments in two strawberry cultivars indicates that *FvCKX1* may promote achene (seed) maturation while potentially repressing receptacle growth and expansion. These observations support an evolutionarily conserved function of *CKX* family genes in the regulation of seed development.

Cytokinins (CKs) play important roles throughout all stages of fruit development. In the fleshy fruit, CKs accumulate in the early stages of fruits, and exogenous CK application has been shown to induce fruit set and promote fruit growth [27,48,57,58,59]. In *Arabidopsis*, trans-zeatin, a major bioactive CK, promotes the juvenile-to-adult phase transition through microRNAs and *TARGET OF EAT1 (TOE1)/TOE2*, an age pathway [60]. Consistent with previous studies in banana and tomato, CK treatment delays the strawberry fruit ripening process as well as achene maturation. Given that CKXs are cytokinin (CK)-degrading enzymes [28], the dynamic changes in CK levels during strawberry fruit development generally exhibited an inverse correlation with *FvCKX1* expression, consistent with the established role of CK in fruit development [55,56,61,62]. While this supports the plausible mechanism of *FvCKX1*-mediated CK catabolism regulating development, trans-zeatin levels remained largely unaltered in *FvCKX1*-overexpressing or knockdown fruits. This discrepancy may arise partly from functional redundancy among *FvCKX* family members (where combinatorial knockdown of multiple isoforms would be required to disrupt zeatin homeostasis), and/or compensatory effects from other bioactive CK species such as iP [28]. In addition, changes in the expression of *FvCKXs* do not necessarily correspond to alterations in their enzymatic activity. Further comprehensive profiling of all CK derivatives in genetically modified fruits would help clarify whether *FvCKX1* regulates strawberry fruit development by modulating cytokinin availability.

*Fragaria vesca* is classified as a non-climacteric fruit, with abscisic acid (ABA) having been well-documented as a key regulator of its ripening process [36,53]. Auxin and gibberellins (GA) were reported to jointly regulate strawberry fruit development and ripening by controlling the transcription of the ABA metabolic gene *FvCYP707A4a* [36]. During early stages, achene-derived auxin and GA maintain high *FvCYP707A4a* expression, keeping ABA levels minimal. As ripening begins, declining auxin and GA reduce *FvCYP707A4a* expression, triggering a feedforward loop that rapidly elevates ABA and initiates the transition from growth to ripening. In later stages, high ABA further suppresses *FvCYP707A4a*, amplifying this regulatory loop [36]. Intriguingly, the cytokinin catabolic enzyme FvCKX1 emerges as a potential novel modulator of this process. The expression of *FvCKX1* positively correlates with *FvCYP707A4a* during strawberry fruit development (Figure 3c) [36], and genetic manipulation of *FvCKX1* reciprocally alters both *FvCYP707A4a* expression and ABA levels, suggesting cytokinin signaling may fine-tune ABA homeostasis during strawberry ripening transition. Since FvCKX1 is a CK catabolic enzyme, all reported functions of CKXs are achieved through modulating CK homeostasis [22,45,63]. Therefore, the observed changes in *CYP707A4a* expression and ABA levels are likely attributed to altered CK levels, given the well-documented antagonistic relationship between CK and ABA in multiple biological processes [64,65]. In *Arabidopsis*, CK and ABA act antagonistically via a core two-component and phosphorylation-based crosstalk network. ABA signaling activates Sucrose non-fermenting 1-Related protein Kinase 2 (SnRK2) kinases, which directly phosphorylate and stabilize type-A ARRs, negative regulators of CK signaling, thereby repressing CK responses. Conversely, CK signaling through type-B ARRs inhibits SnRK2 kinase activity and promotes proteasomal degradation of ABA INSENSITIVE 5 (ABI5), a key ABA-responsive transcription factor, thus antagonizing ABA signaling. Additionally, ABA downregulates CK biosynthesis genes, while CK upregulates *type-A ARRs* that repress *ABI5* expression, forming a reciprocal regulatory loop that balances plant growth and stress adaptation [64,65]. Nevertheless, during strawberry fruit development, whether CK and ABA remain antagonistic to each other remains unclear. While the canonical function of CKXs is cytokinin catabolic activity, a direct regulatory role of *FvCKX1* in ABA biosynthetic gene expression cannot be ruled out. Further investigations using ChIP-seq or DAP-seq may be warranted to elucidate whether *FvCKX1* modulates target genes through non-canonical mechanisms. Therefore, the molecular mechanisms whereby *FvCKX1* regulates *FvCYP707A4a* are still unresolved, representing a critical knowledge gap in our understanding of non-climacteric fruit ripening.

In summary, transcriptomic analyses revealed substantial differences in gene expression patterns between achenes and receptacles, with a substantial number of genes exhibiting opposing expression trends during the maturation transition phase, indicative of coordinated development between these two tissues. Among these differentially regulated genes, *FvCKX1* was found to positively regulate achene maturation while simultaneously inhibiting receptacle expansion based on transient RNAi and overexpression assays. In receptacles, this regulatory effect appears to be mediated, at least partially, through modulation of the ABA catabolic gene *FvCYP707A4a*. Further generation of stably transformed genetic materials and evaluation of diverse CK forms will be necessary to resolve remaining limitations in dissecting the function of *FvCKX1* and the molecular mechanisms by which it governs strawberry fruit development. Nevertheless, the manipulation of CKXs holds significant promise for biotechnological applications, with broad implications for agriculture, horticulture, and agroforestry [63]. Therefore, *FvCKX1* shows strong potential for enhancing strawberry fruit yield and precisely modulating ripening through targeted expression control. Its ABA-mediated effects may additionally enhance stress resilience and fruit quality.

## 4. Materials and Methods

### 4.1. Plant Materials and Growth Conditions

Plants of *Fragaria vesca*, Yellow Wonder (YW 5AF7, white-type fruit) and Rugen (Ru F7-4, red-type fruit), were used in this work. Seedlings were grown in a growth room with 16 h light, 55% relative humidity and 22 °C. Hand pollination was performed on flowers in full bloom to ensure normal fruit development. The flowers fully opening were defined as stage 1 (S1) for 0 day after anthesis (DAA), and then the developing stages of S1–S7 and the ripening stages (RS) of RS1-RS5 were defined as reported [36]. Fifteen to 20 fruits from at least five plants at the appropriate developmental stage were collected and frozen immediately in liquid nitrogen. Then, achenes were removed from receptacles using tweezers. Three biological replicates were prepared for receptacles and achenes respectively.

### 4.2. Hormone Measurements

Fruits from S1 to RS2 stages were used to measure the endogenous phytohormone levels. The active cytokinin of trans-zeatin (tZ) and abscisic acid (ABA) were quantified using achenes and receptacles separately. A total of 15–20 fruits from the five independent plants were collected, then achenes and receptacles were separated after freezing in liquid nitrogen and then ground to powder. 900 μL ethyl acetate and 100 μL of 50 ng/mL [^15^C_4_]-tZ (OlChemIm, Czech Republic) or 50 ng/mL ^2^H_6_-ABA (OlChemIm, Czech Republic) isotope internal standard were added to each sample (200 mg). The samples were vortexed and sonicated for 20 min under 4 °C and centrifuged at 4 °C for 10 min at 14,000× *g*. The supernatant was transferred into a new 2 mL centrifugal tube, then centrifuged to dry. The precipitation was redissolved with 200 μL of methanol. After filtration with 0.22 μm PVDF filter, solution was put in the sample bottle and analyzed by UPLC-QqQ MS (ACQUITY TQD, Waters, USA). Hormones level of tZ and ABA were calculated though isotope internal standard.

### 4.3. RNA Isolation and Gene Expression Analysis

Total RNA was extracted using the polysaccharide and polyphenolics-rich RNAprep Pure kit (Tiangen, China), on-column DNase digestion with the RNase-Free DNase I (Tiangen) was used to remove contaminating DNA. For real-time quantitative PCR, the cDNA was synthesized from 1 μg total RNA using the TransScript^®^ II One-Step gDNA Removal and cDNA Synthesis SuperMix (TransGen, China). Real-time quantitative PCR (qRT-PCR) analyses was performed using CFX96TM Real-Time System with the SYBR^®^ green supermix (Bio-Rad, USA). FvACTIN [36] was used as the internal control. The primer sequences used for the qRT-PCR are listed in Supplementary Table S1.

### 4.4. Generation of the Transcriptome and the PCoA Analysis

An amount of 1–2 ug total RNA for each sample was sent to the Novogene for RNA-seq with three biological replicates. The transcriptome libraries were constructed using NEB Next^®^Ultra™II RNA Library Prep Kit for Illumina^®^ and then sequenced with the Illumina NovaSeq 6000 (150 bp paired-end reads). The sequencing error rate was denoted by e, and then Qphred = −log10(e) was used to evaluate the quality of Illumina sequencing. Approximately 6 G clean bases were generated for each sample. The clean bases were mapped to the *Fragaria vesca* Genome v4.0.a2 (downloaded from the Genome data for Rosacease website at www.rosaceae.org/species/fragaria_vesca/genome_v4.0.a2, accessed on 24 February 2026) using HISAT2 v2.2.1 after adapters and low-quality reads were removed. The unigenes were annotated by BLAST 2.13.0 against Fragaria_vesca_v4.0.a2 proteins and Fragaria_vesca_v4.0.a2 transcripts provided in Rosaceae website. Gene expression levels were counted using featureCounts v2.0.1 [66], and the uniquely mapped reads pairs were filtered with STAR 2.7.6a [67]. The overall comparisons of the expression profiles between samples were performed using PCoA (https://www.omicshare.com, accessed on 24 February 2026). PCoA analyzed the relative enrichment of OTU and the Bray–Curtis in R, then visualized the results using ggplot2 based on the expression values.

### 4.5. Identification of Specially Regulated Genes, Venn Analysis and GO Functional Enrichment Analysis

The Differential Expression analysis for Sequence data 2 (DESeq2) was used to select the differentially expressed genes (DEGs) between stages for achenes and receptacles. Adjusted through the Benjamini–Hochberg method, six groups of DEGs were selected based on |log2(fold change)| ≥ 1.5 and *p*-value ≤ 0.05. Using STEM-Short Time-series Expression miner (http://www.cs.cmu.edu/~jernst/stem, accessed on 24 February 2026), trend analysis was performed to understand the gene regulatory profile of achene and receptacle during fruit ripening transition; the expression values were adjusted using log2(fold change); DEGs were selected with |log2(fold change)| ≥ 1.5, *p*-value ≤ 0.05. The genes specifically involved in fruit development were collected based on expression value > 50. The DEGs identified in the achene and receptacle development and trend analysis were used in the following studies based on the functional enrichment analysis and expression profiling. To explore the tissue-specific gene expression patterns, Venn analysis (https://www.omicstudio.cn/tool/157, accessed on 24 February 2026) was performed to analyze the distribution of DEGs between the receptacle and achene.

Gene Ontology (GO) enrichment analysis was performed based on the GO-annotation database of Fvesca_v4.0.a2_genes2Go, which was downloaded from Rosaceae website (https://www.rosaceae.org/species/fragaria_vesca/genome_v4.0.a2, accessed on 24 February 2026). DEGs identified in the achene and receptacle were analyzed separately. For the similar GO terms with the same genes, only one term was retained based on *p*-value < 0.05 and *Q*-value < 0.05. The enrichment results displayed in barplot, bubble gradient and circular diagram were generated by GraphPad Prism 8 (https://www.Graphpad.Com/scientific-software, accessed on 24 February 2026) and Omicshare Tools (https://www.omicshare.com/tools/Home/Soft, accessed on 24 February 2026).

### 4.6. Phylogenetic Analysis

Gene sequences of the homologs of *CKXs* were downloaded from Rosacease website (https://www.rosaceae.org/search/features, accessed on 24 February 2026) for strawberry and TAIR (https://www.arabidopsis.org/tools/blast/, accessed on 24 February 2026) for *Arabidopsis*. The sequences were aligned using ClustalW, and then, phylogenetic tree was constructed with MEGA 7.0 (https://www.megasoftware.net/, accessed on 24 February 2026) program using the neighbor-joining method and bootstrap analysis (1000 replicates).

### 4.7. Hormone Treatments

S6 fruits were immersed in the hormone solution for 10 s, and treatments were performed every 3 days, then fruit phenotypes were observed before each treatment. To study the effects of hormones on gene function, plant hormone was applied when the injection was performed. In phenotype analysis, the final concentrations of treatments were 500 μM and 100 μM for TDZ (thidiazuron), 100 μM lovastatin (a cytokinin biosynthetic inhibitor), 100 μM ABA (abscisic acid) and 10 mM Na_2_WO_4_ (ABA biosynthetic inhibitor). The solution included 0.1% ethanol and 0.01% Tween 20.

### 4.8. Gene Functional Verification

Functional analysis of *FvCKX1* was performed using the transient transformation system [36,49]. Full-length CDS of *FvCKX1* (1494 bp) was cloned into the pDONR-207 using the BP reaction. Using the LR reaction, the fragment was then recombined into the pK7WG2D gateway destination vector, which carried a p35S promoter-driven eGFP and worked well in strawberry. A 276 bp cDNA fragment which came from 5′ end 0-276 was cloned and inserted into pDONOR-207 using the BP reaction, and the gateway destination vector of pK7WIWG2D was used, which carried a p35S-driven eGFP and worked well in strawberry [11,49]. Primers used for the vector construction are shown in the supplemental datasets.

The constructed vectors of pK7WG2D-FvCKX1 (FvCKX1-OE) and pK7WIWG2D-FvCKX1 (FvCKX1-RNAi) were introduced into *Agrobacterium tumefaciens* (GV3101) by electroporation, and the transformed GV3101 transiently expressed the vectors in the tobacco leaves. After 48 h, GFP signal was checked in the tobacco leaves infiltrated with GV3101 to verify the vectors’ function properly. Verified Agrobacterium strain GV3101 harboring pK7WG2D-FvCKX1 (*FvCKX1*-OE) or pK7WIWG2D-*FvCKX1* (*FvCKX1*-RNAi) was cultured at 28 °C in LB medium. After 24 h, bacterial cells were harvested and resuspended in infiltration buffer (10 mM MgCl_2_, 10 mM MES, pH 5.6, 150 mM acetosyringone) to a final OD_600_ of 1.0, then shaken in the dark at room temperature for 2–3 h prior to infiltration. Each Agrobacterium strain was subsequently injected into the carpopodium of S6-stage strawberry fruits still attached to the plant using a 1 mL needle-less syringe, following a previously described method [36]. The GFP signal was checked to verify the efficiency of transformation system. To confirm the function of FvCKX1-OE and FvCKX1-RNAi, fruits in S6, S7, RS1 and RS2 stages were characterized. A total of seven to 10 fruits from five independent plants that showed GFP signal were used for the experiments. The gene expression variations were checked 8 days after injection.

### 4.9. Statistical Analysis

All fitted curve and bar graphs were generated by GraphPad Prism 8 software (https://www.graphpad.com/scientific-software, accessed on 24 February 2026). Statistical significances were also performed using Student *t* test and/or one-way ANOVA.

## Data Availability

All data generated or analyzed during this study are included in the. article and its Supplementary Materials.

## Supplementary Materials

The following supporting information can be downloaded at: https://www.mdpi.com/article/10.3390/plants15081171/s1, Figure S1: The differentially expressed genes (DEGs) identified in receptacle (Rec) and achene (Ach) during the ripening transition stages of S6 to RS1; Figure S2: Gene expression patterns of the five well-characterized regulatory programs during fruit ripening transition; Figure S3: Detailed development of red-type fruit; Figure S4: Fruit growth response to treatment with a cytokinin biosynthesis inhibitor; Figure S5: Development of red-type fruit in respond to cytokinin and cytokinin biosynthesis inhibitor treatments; Figure S6: Effect of *FvCKX1* on Zeatin content variation in the receptacle of *Fragaria. vesca*; Figure S7: The endogenous abscisic acid (ABA) content in the receptacle and achene; Table S1: Primers used for qRT-PCR; Table S2: Primers used for vector construction; Table S3: Accession ID.
