# Supplementary material for: Cytokinin Oxidase/Dehydrogenase 1 (FvCKX1) Coordinates Receptacle Growth and Achene Maturation in Strawberry
Source: Plants (Basel). 2026 Apr 10;15(8):1171. doi: 10.3390/plants15081171 (PMC13120239; doi:10.3390/plants15081171)
Supplement: Supplementary file 1 [file plants-15-01171-s001.zip › plants-4197716-supplementary.pdf]

Supplementary data

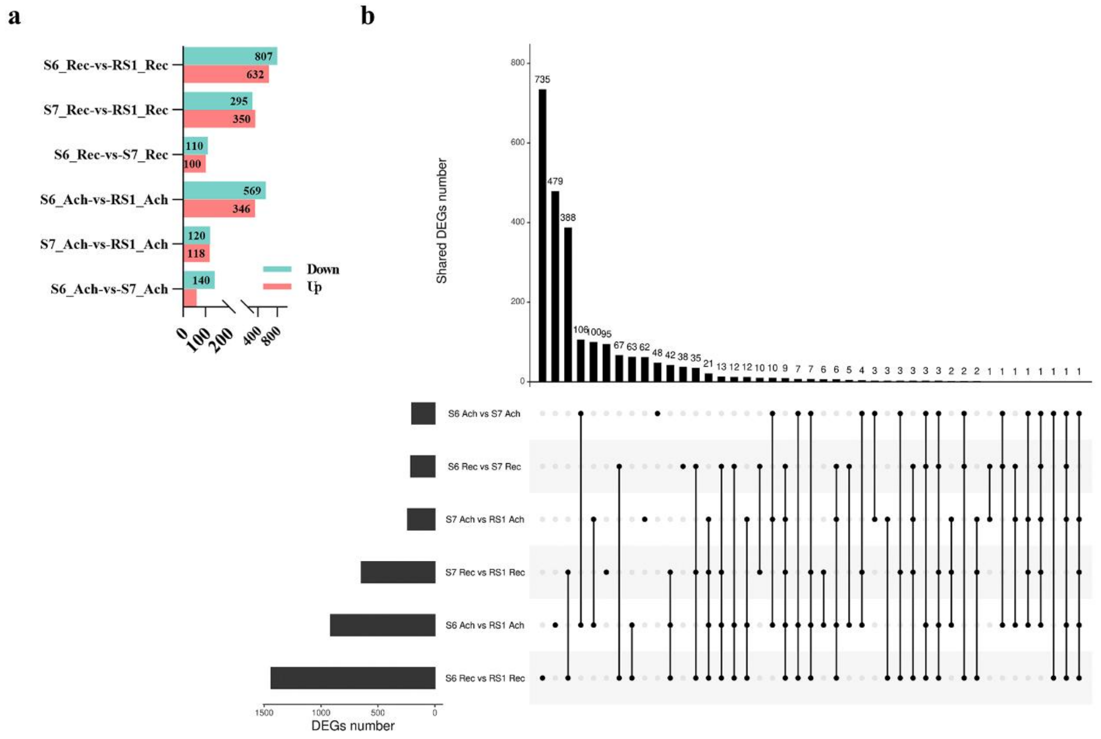

Supplementary Figure S1. The differentially expressed genes (DEGs) identified in receptacle (Rec) and achene (Ach) during the ripening transition stages of S6 to RS1.

(a) Histogram showed the DEGs identified from the receptacle and achene respectively. The numbers of up- or down-regulated genes were labeled in different color. (**b**) Venn diagram showed the overall results of the DEGs identified from the achene and receptacle during S6-RS1. The groups were labeled on the right. Histogram showed the number of genes in each group.

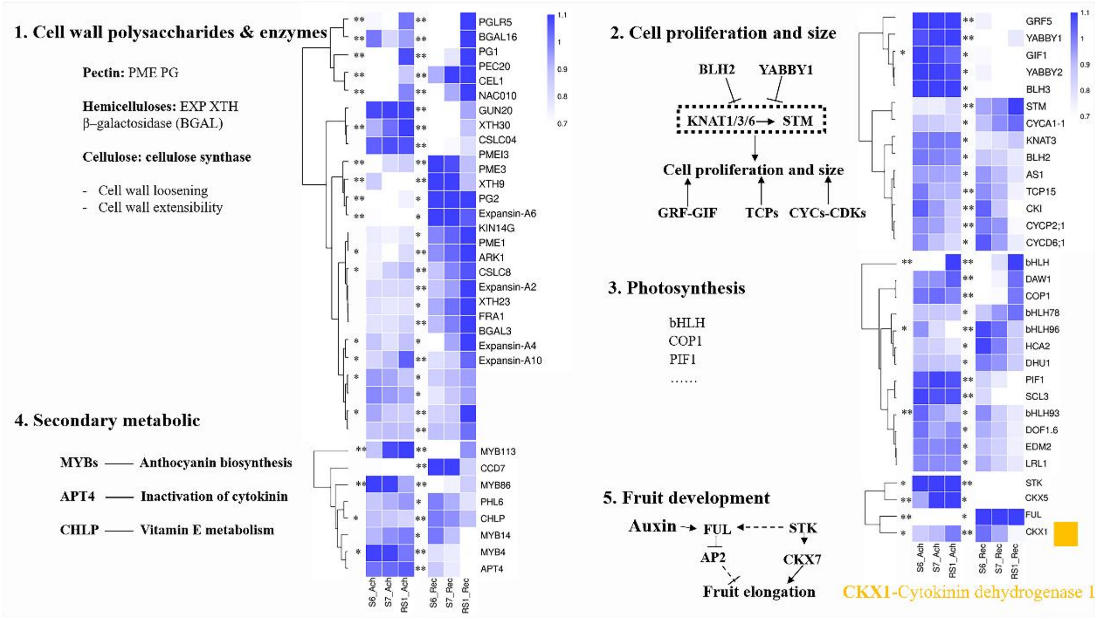

Supplementary Figure S2. Gene expression patterns of the five well-characterized regulatory programs during fruit ripening transition.

Five regulatory programs associated with different developmental events are shown to the left of heatmaps. Heatmaps show the gene expression patterns in the S6-RS1 achene and receptacle. Expression value = Log2(value+10^{-6}). Significant differences between groups are labeled (p < 0.05, one way ANOVA). The gene marked in orange shows that FvCKX1 is significantly regulated.

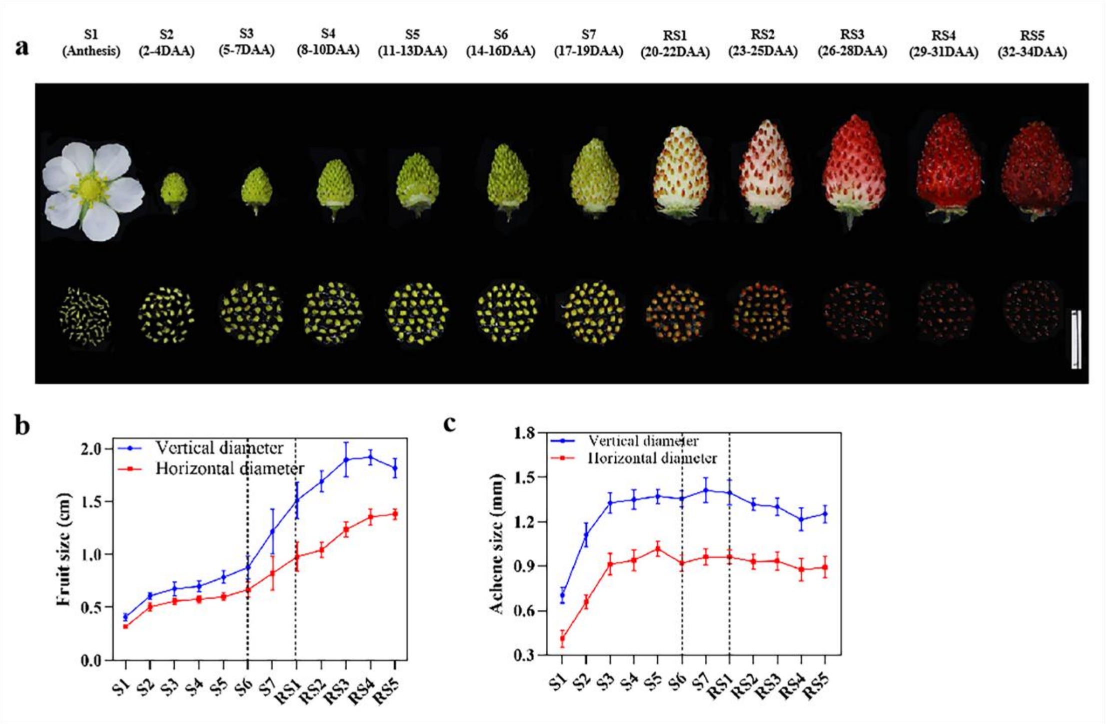

Supplementary Figure S3. Detailed development of red type fruit.

(**a**) Detailed description of the different developmental stages of the red type *Fragaria. vesca*. fruit. The different growth stages were distinguished by the wide-type fruit. Achenes were separated from the receptacle. Scale bar =1 cm. The size of fruits (**b**) and achenes (**c**) (vertical diameter and horizontal diameter) were quantified. Error bars represent SD, n = 15-20 in (b). Error bar represents SD, n = 60 in (c).

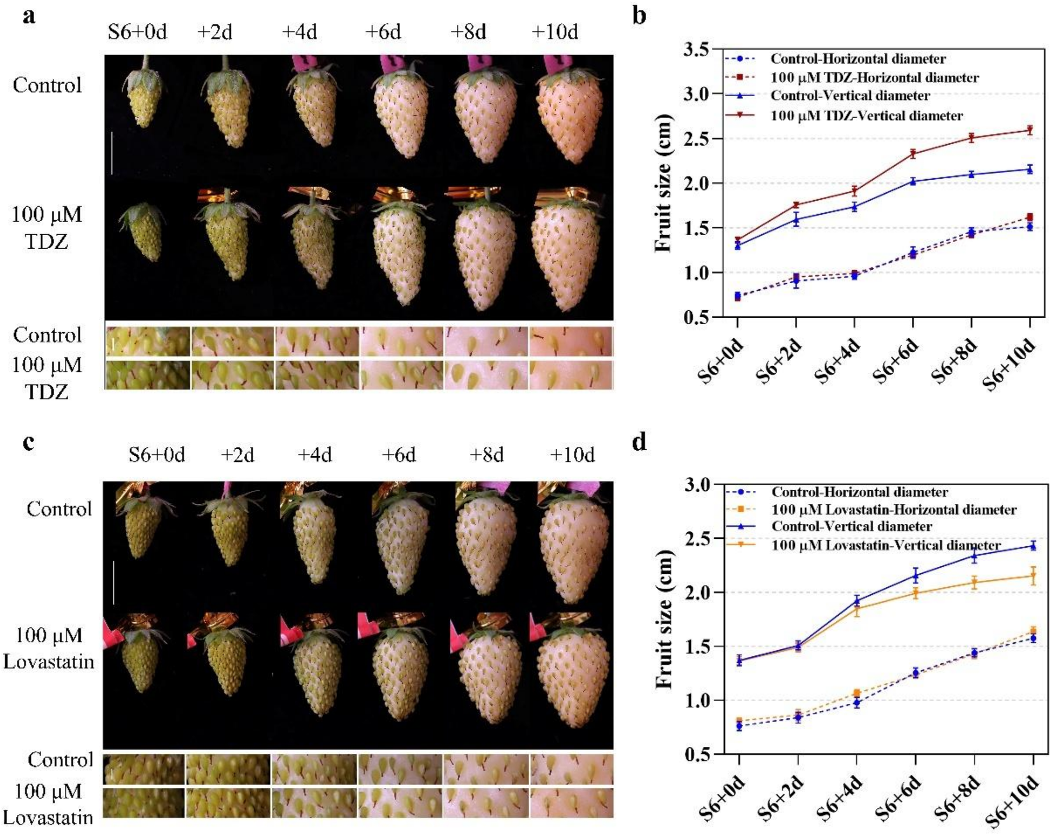

Supplementary Figure S4. Fruit growth response to treatment with a cytokinin biosynthesis inhibitor.

(**a-b**) Receptacle expansion can be promoted by 100 µM TDZ, while achene senescence was inhibited. Fruit dimensions (length and width) were quantified in (b). Fruits were characterized every 2 days. Error bars represent SD of 7-10 fruits.

(**c-d**) Receptacle expansion can be promoted by 100 µM Lovastatin, while achene senescence was inhibited. Fruit dimensions (length and width) were quantified in (d). Fruits were characterized every 2 days. Error bars represent SD of 7-10 fruits. Lovastatin is cytokinin biosynthesis inhibitor.

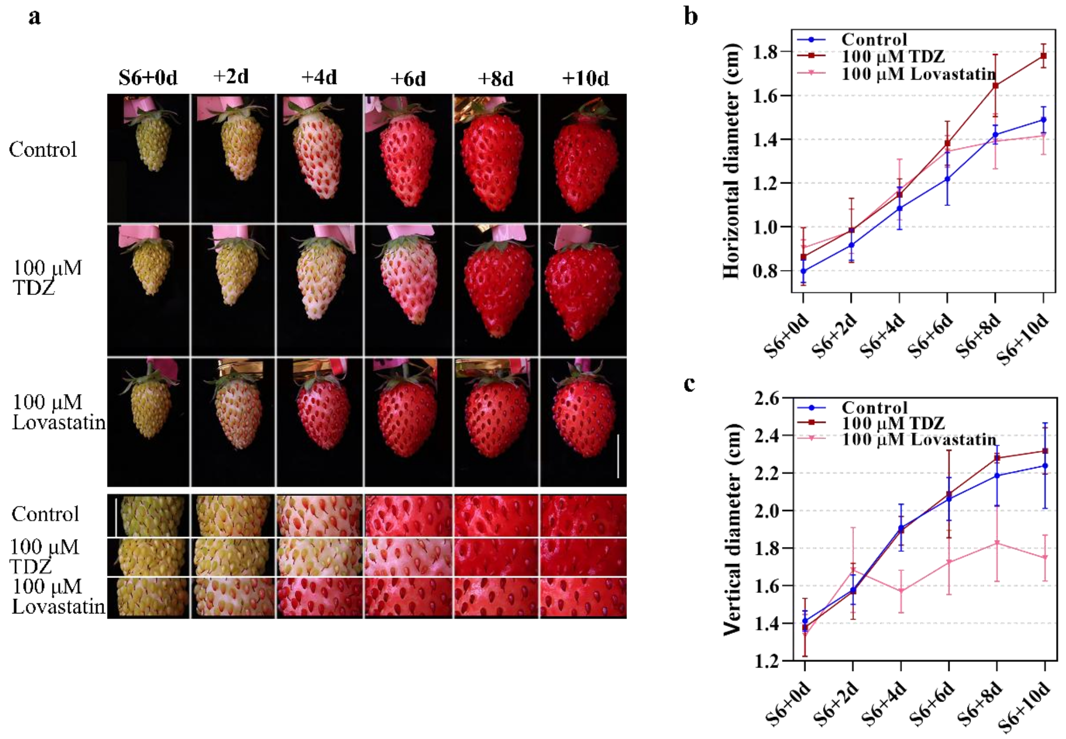

Supplementary Figure S5. Development of red-type fruit in respond to cytokinin and cytokinin biosynthesis inhibitor treatments. Quantification of fruit growth in respond to TDZ or lovastatin treatment. Scale bar = 1 cm for whole fruits. Scale bar = 0.5 cm for the enlarged view. Fruit size variation in response to different treatments was shown as changes in horizontal diameter (**b**) and vertical diameter (**c**). Error bars represent SD, n = 7-10.

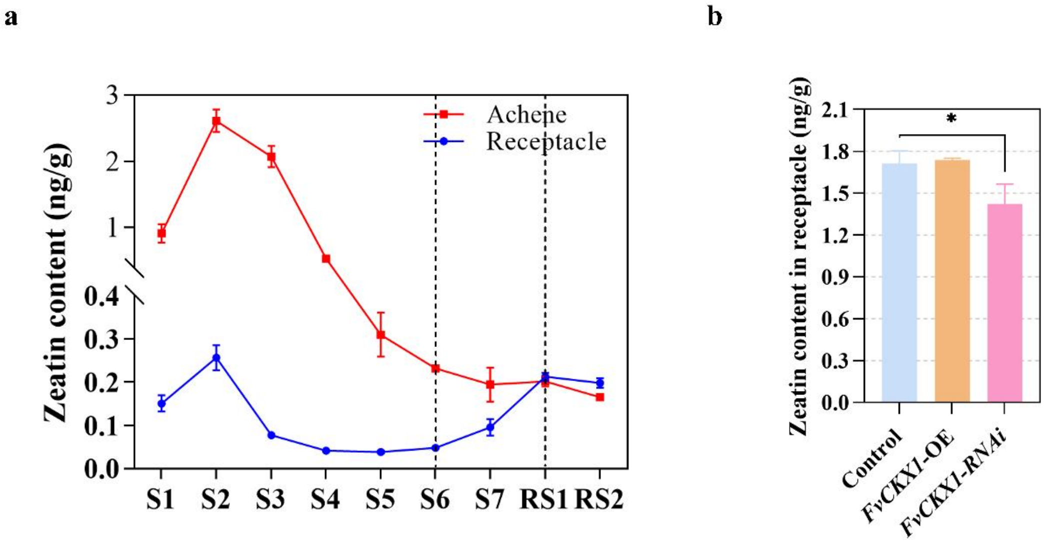

Supplementary Figure S6. Effect of *FvCKX1* on Zeatin content variation in the receptacle of *Fragaria. vesca.* (**a**) Dynamic changes of endogenous cytokinin (trans-Zeatin, Zeatin) content in the receptacle and achene during stages S1 to RS2. Error bars represent the SD from three independent repeats. n = 15-20 fruits. (**b**) Zeatin content in *FvCKX1* OE and *RNAi* receptacles. Error bars represent SD of three independent repeats. One-way ANOVA for statistical analysis; *, $p < 0.05$.

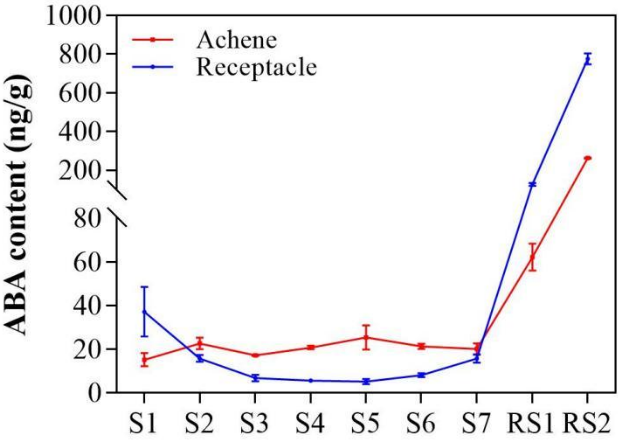

Supplementary Figure S7. The endogenous abscisic acid (ABA) content in the receptacle and achene. Error bars represent the SD from three independent repeats. n = 15-20 fruits.

Supplementary Table S1. Primers used for qRT-PCR.

Gene name	Forward primer (5'-3')	Reverse primer (5'-3')
FvACTIN	CAGAAAGATGCTTATGTCGG	TGGGGCAACACGAAGCTCAT
FvCKX1	CCACACCCATGGCTCAATCT	TAGGGCCGTTGCTTGTTTCT
FvCKX2	CTCGAGGAGTGTTCGGCAAT	CCGGTGTAACCACGGAAGTT
FvCKX3	GCATGGACCCCAGATCAACA	ATGCCAAACTGTCCTAGCCC
FvCKX4	CTCCCCAAACAACAACGCAG	CCCACTTGACCCTTGTTGGT
FvCKX5	ACGCAGCTGCAGTTCTGTAT	GCGACACCACAATTCGTGAC
FvCKX6	AGCTTGAAGTCGTTACGGGG	TCGAGCTCTGGTGATGATGC
FvCKX7	GACTTCCAAAGGAAGCCGGA	ACGAATCCTAAGCCTCGCAG
FvCKX8	CTGGGAAACAAAACCAGCGG	CCGACGGTTCTGATTGGTGA
FvPG1	TCTCATGCGCAACAGGGAAT	GTTGCGGCCGAATCCAATAC
FvPEC21	ATCGGAACCGGAGACGATTG	CGTACCTTCCGAGACTTCCG
FvCEL1	TATAGACTTCGGGCGGGTCA	CCCAGCAGTTATGGTCGGAG
FvABI4	CCTCTTCGGCTCATCCTTA	TGTGGCTGGTGTACTTGGTAT
FvNCED3	CGGAATCAGATGGGTCGAGG	GACGTGTCGACTTCCCTGTT
FvNCED5	CTGTCCGTCATTCACTCCCT	CACCGTCATTGATGCTAACG
FvCYP707A1/3	ATCTTGGGATGTCCTTGCG	TGCGTGGTAGTCTCCTTGGT
FvCYP707A2	CTTTCACCAGGGAGATTACCA	CAAGAAAGCCGACATCAAACAC
FvCYP707A3	TCAGTGTGTTCATGACCGGG	AAGCTGGTTGGACCTAGCAC
FvCYP707A4a	CCCGCTACAAAGAAGAACTGA	TGTCACCGATAATGTGCCTC
FvCYP707A4b	ACTTGGCTGTCCTTGTGTCA	ACTGTGGTAATCTCCCTGGTG
FvPYL2	TCTGGATGACGAGAAGCACG	CCGAGTAAACCTTGCCGTCT

Supplementary Table S2. Primers used for vector construction.

Gene name	Sequence for primer (5'-3')	Vector
FvCKX1 OE F	ATGCCTTCATTAAACACAC	pK7WG2D.1
FvCKX1 OE R	TGAGAAGGATATTGCCTT	
FvCKX1 RNAi F	ATGCCTTCATTAAACACACTTCCAG	pK7GWIWG2
FvCKX1 RNAi R	GGGCCGCTTAAGTGATTCCA	

Supplementary Table S3. Accession ID.

Gene	Accession ID	Gene	Accession ID	Gene	Accession ID
FvCKX1	FvH4_7g02150	KIN-4A	FvH4_2g33960	GPT1	FvH4_3g02500
FvCKX2	FvH4_3g03260	SBT19	FvH4_2g34170	HPCA1	FvH4_3g23870
FvCKX3	FvH4_6g24620	PP2C12_ARATH	FvH4_2g34570	ACCH5	FvH4_4g03310
FvCKX4	FvH4_1g07620	OPT3	FvH4_3g00400	NUD17	FvH4_4g33680
FvCKX5	FvH4_1g07610	GPDL2	FvH4_3g13670	Hypothetical protein	FvH4_4g35710
FvCKX6	FvH4_2g30990	Hypothetical protein 1	FvH4_3g16010	NRT1.1	FvH4_5g00150
FvCKX7	FvH4_3g04610	RABA1B	FvH4_3g18190	BT1	FvH4_5g06740
FvCKX8	FvH4_2g39230	Hypothetical protein 2	FvH4_3g21110	ELP3	FvH4_5g07410
AtCKX1	AT2G41510	BB2	FvH4_3g29040	PHD6	FvH4_5g13940
AtCKX2	AT2G19500	EF1A	FvH4_3g33060	CYP75B1	FvH4_5g14010
AtCKX3	AT5G56970	PUM12	FvH4_3g45790	At1g72190	FvH4_5g31330
AtCKX4	AT4G29740	LBO1	FvH4_4g25590	LAC14	FvH4_5g35760
AtCKX5	AT5G21482	C2H2-like	FvH4_4g30350	PUP3	FvH4_5g36040
AtCKX6	AT1G75450	DNAJ1	FvH4_4g32391	PRP1	FvH4_5g36071
AtCKX7	AT3G63440	DRP1C	FvH4_4g33500	PHL6	FvH4_6g36430
FvPG	FvH4_6g41380	PEM	FvH4_4g33960	CKX1	FvH4_7g02150
FvPEC20	FvH4_2g19540	GEML6	FvH4_4g37190	RLP35	FvH4_7g10111
FvCEL1	FvH4_4g33940	At1g63300	FvH4_5g02001	NIK1	FvH4_7g22640
ITPK4	FvH4_1g00201	F-box protein	FvH4_5g08330	EDM2	FvH4_4g08730
TMN10	FvH4_1g00890	TSN1	FvH4_5g09960	DOF1.6	FvH4_2g40660
RNA polymerase	FvH4_1g05980	hypothetical protein 3	FvH4_6g14750	HCA2	FvH4_5g05330
RING/U-box	FvH4_1g19860	ERG24	FvH4_6g17830	PIF1	FvH4_2g19790
SCP18	FvH4_2g03523	ATA4	FvH4_6g24590	DAW1	FvH4_1g10310
GASA7	FvH4_2g04680	ROD1	FvH4_6g29670	DHU1	FvH4_1g17060
PRF4	FvH4_2g26231	FAD5	FvH4_6g29730	COP1	FvH4_3g01260
UGT74E1	FvH4_1g00400	HAD superfamily	FvH4_6g32560	bHLH93	FvH4_1g18930
UBC37	FvH4_1g04830	PHO19	FvH4_6g48060	bHLH96	FvH4_5g36240
IST1L	FvH4_1g10740	NRT2.7	FvH4_6g51160	bHLH93	FvH4_5g33810
MYB73	FvH4_1g16770	ATL70	FvH4_7g01050	LRL1	FvH4_1g01110
NIR	FvH4_2g05960	WEI2	FvH4_7g24230	bHLH78	FvH4_4g12760
TAR4	FvH4_2g13010	SKS1	FvH4_7g27730	bHLH	FvH4_4g07800
GLR2.8	FvH4_2g37160	MYB14	FvH4_2g21500	SCL3	FvH4_7g02290
FRA1	FvH4_2g33960	PHL6	FvH4_6g36430	MYB113	FvH4_1g22020

KIN14G	FvH4_2g14960	APT4	FvH4_5g00190	MYB86	FvH4_5g06070
ARK1	FvH4_7g18790	NAC010	FvH4_3g08490	PGLR5	FvH4_1g03310
PME3	FvH4_6g35830	Expansin-A2	FvH4_7g25860	KNAT3	FvH4_2g28300
PMEI3	FvH4_1g09220	XTH23	FvH4_3g00850	GIF1	FvH4_6g02460
BGAL3	FvH4_1g12350	XTH9	FvH4_6g43860	GRF5	FvH4_6g34730
BGAL16	FvH4_2g27470	XTH30	FvH4_5g23180	BLH2	FvH4_1g14320
GUN20	FvH4_5g03220	CSLC8	FvH4_6g50280	BLH3	FvH4_2g39260
Expansin-A4	FvH4_3g36410	CSLC04	FvH4_6g52870	CKI	FvH4_2g06360
Expansin-A10	FvH4_4g31140	AS1	FvH4_2g38350	CYCP2;1	FvH4_6g43551
Expansin-A6	FvH4_6g13610	YABBY1	FvH4_5g00320	CYCD6;1	FvH4_6g44360
STM	FvH4_6g07460	YABBY2	FvH4_3g26130	CYCA1-1	FvH4_2g27580
TCP15	FvH4_4g31520	CCD7	FvH4_7g10450	MYB4	FvH4_2g01320
CHLP	FvH4_5g14870	STK	FvH4_5g32540	PME1	FvH4_6g35820
PG2	FvH4_4g34470	FUL	FvH4_5g08530		
